# Prevalence of multidrug-resistant and extensively drug-resistant phenotypes of Gram-negative bacilli isolated in clinical specimens at *Centre Hospitalo-Universitaire* Ibn Rochd, Morocco

**DOI:** 10.11604/pamj.2023.45.41.34457

**Published:** 2023-05-17

**Authors:** Imane Zalegh, Laila Chaoui, Fakhreddine Maaloum, Khalid Zerouali, Rajaa Ait Mhand

**Affiliations:** 1 Research Unit Microbiology, Biomolecules and Biotechnology, Laboratory of Chemistry-Physics and Biotechnologies of Biomolecules and Materials, Faculty of Sciences and Technologies (FST), Mohammedia Hassan II University of Casablanca, Casablanca, Morocco,; 2 Laboratory of Microbiology, *Centre Hospitalo-Universitaire (CHU)* Ibn Rochd, Casablanca, Morocco

**Keywords:** *Enterobacteriaceae*, *Acinetobacter baumannii*, *Pseudomonas aeruginosa*, antimicrobial resistance, CTX-M, OXA-48, Morocco

## Abstract

**Introduction:**

antimicrobial resistance in gram-negative bacilli is one of the major concerns in public health. We aimed to evaluate gram-negative bacilli epidemiology, antimicrobial profiles, and the resistance´s mechanism for Enterobacteriaceae isolated from specimens of hospitalized patients in wards of University Hospital Center Ibn Rochd of Casablanca, Morocco.

**Methods:**

a prospective study of the patient's specimens, collected from December 2016 to 31^st^ March 2017. Isolation and identification were performed using conventional biochemical tests. According to the European Committee on Antimicrobial Susceptibility Testing guidelines, antibiotic susceptibility was determined. Polymerase Chain Reaction (PCR) was used to detect β-lactamase and carabapenemase genes: CTX-M, SHV, TEM, OXA-48, NDM, and VIM among the Enterobacteriaceae.

**Results:**

according to inclusion criteria, 38 Enterobacteriaceae, 25 Acinetobacter baumannii (A. baumannii), and 10 Pseudomonas aeruginosa (P. aeruginosa) were included during the study period; these bacteria were mainly responsible for bacteremia. Fifty-five percent of enterobacteria were extended-spectrum β-lactamase (ESBL), 42% EBSL and carbapenemase, and 3% carbapenemase, with high coresistances. Eighty-four percent of A. baumannii were XDR. All P. aeruginosa were MDR; amikacin showed the best activity (70% susceptibility). The genotypic approach revealed the presence of bla_CTX-M_, bla_SHV_, bla_TEM_ in 68%, 22%, and 11% respectively. Of the 22 carbapenemase-producers, 41% were bla_OXA-48_ and 18% bla_NDM_; none had bla_VIM_. Furthermore, various genes coexistence were detected: bla_CTX-M_+bla_OXA-48_; bla_CTX-M_+bla_NDM_; bla_CTX-M_+bla_SHV_+bla_OXA-48_; and bla_SHV_+bla_OXA-48_.

**Conclusion:**

findings revealed highly resistance rate among isolates. This raises the need to control antibiotics and regular screening to identify dynamics promoting resistance. Thus, we recommend developing antimicrobial stewardship programs and improving hygiene systems to prevent the nosocomial spreading of these phenotypes in our center.

## Introduction

Bacteria are one of the primary sources of infectious diseases in humans. Nowadays, gram-negative bacilli, including *Enterobacteriaceae, Acinetobacter baumannii* (*A. baumannii*), and *Pseudomonas aeruginosa* (*P. aeruginosa*), have been identified as the primary cause of infections. Therefore, these pathogens are becoming challenging in developing and developed countries significantly among hospitalized and non-hospitalized patients. Furthermore, they are increasingly isolated in different hospital wards from respiratory tract infections, urinary tract infections, intra-abdominal infections, septic, post-surgical infections, and pneumonia, a severe menace, especially in critically ill patients [1,2].

The injudicious misuse and accessibility of antibiotics have led to the emergence of antimicrobial-resistant strains to commonly prescribed antibiotics, a significant challenge in medical practice. Antimicrobial resistance (AMR) in this group has been recognized by World Health Organization (WHO) as a threat to the public health [3]. Studies conducted in Morocco have reported the alarming rate of β-lactamase and carbapenemase-producing bacterial pathogens and the increasing prevalence of resistance to other antibiotics classes [4,5]. This problem is crucial in fermenting and non-fermenting bacteria because of their ubiquity and ability to acquire genes that confer resistance to other antibiotics classes. This situation is associated with therapeutic impasses leading to high morbidity and mortality rates and an extended hospital stays. It has been predicted that if appropriate control and prevention measures are not taken, in 2050, AMR would be responsible for a death every 3 seconds [6]. Accordingly, in the current study, we established the period prevalence and antimicrobial sensitivity profile of the following phenotypes ESBL and carbapenemase-producing of the main pathogenic bacteria *Enterobacteriaceae, A. baumannii P. aeruginosa* imipenem resistants. Then we detected the presence of β-lactamase and carbapenemase encoding genes: *bla*_CTX-M_, *bla*_SHV_, *bla*_TEM_, *bla*_OXA-48_, *bla*_NDM_, and *bla*_VIM_among the *Enterobacteriaceae* isolated from various clinical specimens from hospitalized patients at University Hospital Center, Ibn Rochd of Casablanca, Morocco.

## Methods

**Study design:** this is a descriptive cross-sectional study, conducted between December 2016 and March 2017 in University Hospital Center Ibn Rochd of Casablanca, Morocco. Data was taken from the computerized database of the Microbiology Laboratory. The inclusion criteria were *Enterobacteriaceae* ESBL and/or carbapenemase-producing, *A. baumannii*, and *P. aeruginosa* imipenem resistant.

**Study population:** the study concerned all services of University Hospital Center Ibn Rochd of Casablanca, Morocco. It´s a tertiary hospital covering the entire region of Grand Casablanca. Isolated were recovered from urine, pus and wound swab, pleural fluid endotracheal tubes, and blood from patients hospitalized in different hospital wards. Data about patients were composed of age, gender, hospitalized ward, bacteria isolated, and type of clinical specimen.

**Isolate collection and identification:** during the study period, 851 clinical isolates were recovered from different samples received. Two hundred and sixty MDR were identified; among them, 73 non-duplicated strains were included in this cross-sectional study. Isolates were identified based on conventional biochemical tests. In brief, specimens were inoculated to MacConkey agar plate and incubated at 37°C for 18h to 24h. Microscopical examination of gram-stained smears confirmed identity. Bacterial colonies were subjected to conventional biochemical tests, including oxidase, catalase, API 20E, and API 20NE (BioMérieux®).

**Antimicrobial sensitivity testing and ESBL production:** the antimicrobial susceptibility profile of isolates was determined using the disc diffusion method, and the results were interpreted according to the European Committee on Antimicrobial Susceptibility Testing (EUCAST) guidelines [7]. The following antibiotics were used; ampicillin (AMP) (10 μg), amoxicillin/clavulanic acid (AMC) (20/10 μg), piperacillin/tazobactam (PTZ) (30/6 μg), cefepime (CPM) (30 μg), cefotaxime (CTX) (5 μg), ceftazidime (CAZ) (10 μg), ceftriaxone (CRO) (30 μg), meropenem (MEM) (10 μg), ertapenem (ETP) (10 μg), imipenem (IMP) (10 μg), gentamicin (GM) (30 μg), ciprofloxacin (CIP) (5 μg), levofloxacin (LEV) (5 μg), amikacin (AK) (30 μg), tobramycin (TN) (10 μg), netilmicin (NET) (10 μg), fosfomycin (FOS) (200 μg), trimethoprim/sulfamethoxazole (TSU) (1.25/23.75 μg), chloramphenicol (CHL) (30 μg) (Oxoid Ltd, Basingstoke, UK).

All *Enterobacteriaceae* isolates with an inhibition diameter ≤ to 22 mm with CAZ and ≤ to 27 mm with CTX were subjected to the ESBL confirmatory test using the double-disc synergy [8]. In addition, *Escherichia coli* ATCC 25922 and *P. aeruginosa* ATCC 27953 were used as quality control for media and antibiotics performance.

The definition of Magiorakos *et al*. [9] was used to classify bacterial isolates into MDR, extensively drug-resistant XDR, and pan drug-resistant pandrig resistance (PDR). MDR=resistant to at least one agent in three or more antimicrobial classes, XDR= susceptible to two or fewer antimicrobial classes, and PDR=resistant to all antimicrobial agents in all antimicrobial classes.

**Preparation of deoxyribonucleic acid (DNA) and PCR:** polymerase chain reaction technique was used to target the *bla*_CTX-M_ [10], *bla*_SHV_ [11], *bla*_TEM_ [12], *bla*_OXA-48_ [13], *bla*_NDM_ [14], and *bla*_VIM_ [14] among all *Enterobacteriaceae* ESBL and carbapenemase-producing included. All *Enterobacteriaceae* ESBL and carbapenemase-producing were grown on Mueller-Hinton (MH) agar plates (Bio-Rad®) for 18-24 hours at 37°C. Bacterial cells were suspended in 400 μL of ultrapure water. The suspension was heated at 100°C for 10 minutes and immediately frozen at 0°C for 5 minutes. One hundred and eighty μL were then recovered after centrifugation of 12000 g for 10 min. The supernatant containing DNA was stored at -20°C until further use.

Extended-spectrum β-lactamase (ESBL)-producing isolates were screened by PCR simplex for the following β-lactamase-encoding genes: *bla*_CTX-M_, *bla*_SHV_, and *bla*_TEM_ as described by [12]. Also screened by PCR for the following carbapenemase-encoding genes: *bla*_OXA-48_, *bla*_NDM_, and *bla*_VIM_as described by [13] with slight modifications. The primers used for PCR are listed in Table 1.

**Table 1:** primers for polymerase chain reaction (PCR) amplification of genes mediated resistance

Genes	Primers	Sequences (5'-3')	Products size (pb)
*bla*_CTX-M_	CTX-M1-F	TTGGTGACGATTTTAGCCGC	864
	CTX-M1-R	GGT TAA AAA ATC ACT GCG TC	
*bla*_SHV_	SHV-F	TTATCTCCCTGTTAGCCACC	870
	SHV-R	GATTTGCTGATTTCGCTCGG	
*bla*_TEM_	TEM-F	ATA AAA TTC TTG AAG ACG AAA	1080
	TEM-R	GAC AGT TAC CAA TGC TTA ATC A	
*bla*_OXA_	OXA-48-F	GCTTGATCGCCCTCGATT	281
	OXA-48-R	GATTTGCTCCGTGGCCGAAA	
*bla*_NDM_	NDM-F	GGTTTGGCGTCTGGTTTTC	621
	NDM-R	CGGAATGGCTCATCACGATC	
*bla*_VIM_	VIM-F	GATGGTGTTTGGTCGCATA	390
	VIM-R	CGAATGCGCAGCACCAG	

For *bla*_CTX-M_, *bla*_SHV_, and *bla*_TEM_ amplification mixture were performed in volume of 23.5 μL containing: 2.5 μL of MgCl_2_(2.5 mM), 5 μL of buffer, 0.5 μL of each forward and reverse primers (0.2 μM), 0.5 μL of deoxynucleoside triphosphate (dNTP) (0.2 mM), 14.3 μL of double-distilled water (ddH_2_O), and 0.2 μL (1 unit) of Taq DNA polymerase. Then we added 1.5 μL of the bacterial DNA for each simplex PCR.

For *bla*_OXA-48_ amplification mixture was performed in volume of 22 μL containing: 2.5 μL of MgCl_2_(2.5 mM), 5 μL of buffer, 0.5 μL of each forward and reverse primers (0.2 μM), 0.5 μL of dNTP (0.2 mM), 12.8 μL of ddH_2_O, and 0.2 μL (1 unit) of Taq DNA polymerase. Then we added 3 μL of the bacterial DNA. Amplification reactions for *bla*_NDM_ and *bla*_VIM_ were performed in a volume of 22 μL containing: 2.5 μL of MgCl_2_(2.5 mM), 5 μL of buffer, 1 μL of each forward and reverse primers (0.2 μM), 2 μL of dNTP (0.2 mM), 10.3 μL of ddH_2_O, and 0.2 _µ_L (1 unit) of Taq DNA polymerase. Then we added 3 μL of the bacterial DNA. Amplicons were visualized after running at 120V for 30 min on a 0.5% agarose gel containing ethidium bromide (0.5 μg/mL), using photographed ultraviolet (UV) transilluminator and analyzed compared with the positive control of each resistance gene and DNA Ladder 100 bp (Promega®).

**Data analysis and interpretation:** the data was collected and entered into Microsoft Excel 16.59. The frequency and percentages of MDR, carbapenemase, and ESBL-producing gram-negative bacteria were calculated using SPSS®-26. Tables were used for data presentation.

**Ethical considerations:** in order to ensure the confidentiality of patient´s personal information, data of isolates recovered during the diagnosis routine were collected and anonymity was maintained. So, no personal information of patients was taken into consideration in this study.

## Results

**Demographic characteristics of patients with bacterial infections:** out of 851 different clinical cultures were submitted to the laboratory during the specified period. Of these isolates, 626 (73.5%) *Enterobacteriaceae*, 101 (11.9%) *A. baumannii*, 124 (14.6%) *P. aeruginosa*. Among the enterobacteria isolated, 147 (24%) were multi-resistant. For gram-negative non-fermentative bacteria, 88 (87%) and 25 (20%) were Imipenem resistant for *A. baumannii* and *P. aeruginosa*, respectively. According to our inclusion criteria, 38 (52%) enterobacteria, 25 (34%) *A. baumannii* imipenem resistant, and 10 (14%) *P. aeruginosa* imipenem resistant was included. The distribution of 73 isolates according to specimen types showed the highest proportion of isolates, were from blood 24/73 (33%), followed by urine 14/73 (19%), 9/73 (12%) rectal swabs, 9/73 (12%) pus, 9/73 (12%) medical devices, 7/73 (9%) clinical respiratory specimens, and 1/73 (1%) from body fluid (Table 2). The number of isolates was distributed in the wards as follows; haematology: 18/73 (25%), neonatology: 18/73 (24%), intensive care unit (ICU): 16/73 (20%), burned centre 4/73 (5%), urology: 4/73 (5%), visceral surgery: 1/73 (1%), endocrinology and metabolic diseases: 2/73 (3%), day hospital collecting centre: 2/73 (3%), medical pediatrics: 2/73 (3%), orthopedic-trauma centre: 2/73 (3%), physical medicine and functional rehabilitation 1/73 (1%), hepato-gastro-enterology and proctology: 1/73 (1%), infectious diseases: 1/73 (1%), and dermatology: 1/73 (1%) (Table 2).

**Table 2:** distribution of isolates among clinical specimens and wards hospital

	Isolates (N)							
**Species**	***E. coli***	***K. pneumoniae***	***E. cloacae***	***K. oxytoca***	***E. aerogenes***	***R. terrigena***	***A. baumannii***	***P. aeruginosa***
**Specimen**								
Blood	7	6	1	-	-	1	7	2
Urines	6	2	1	1	-	-	2	2
Pus	3	-	1	-	-	-	4	1
Medical equipment	-	-	-	-	-	-	9	-
Bronchial specimens	-	-	-	-	-	-	3	4
Body fluid	-	-	-	-	-	-	-	1
Rectal swab	3	4	1	-	1	-	-	-
Total	19	12	4	1	1	1	25	10
**Wards**								
Hematology	10	3	2	-	1	1	1	-
Neonatology	-	5	-	-	-	-	13	-
Medical ICU	-	1	-	-	-	-	8	7
Burned center	-	1	-	-	-	-	-	3
Urology	3	-	-	-	-	-	1	-
Visceral surgery	1	-	-	-	-	-	-	-
EMD	2	-	-	-	-	-	-	-
M. Pediatrics	1	-	-	1	-	-	-	-
OTC	-	-	1	-	-	-	1	-
PMFR	-	-	1	-	-	-	1	-
HGEP	1	-	-	-	-	-	-	-
IDD	1	-	-	-	-	-	-	-
Dermatology	-	-	-	-	-	-	1	-
DHCC	-	2	-	-	-	-	-	-

EMD: endocrinology and metabolic diseases; M. Pediatrics: medical pediatrics; OTC: orthopedic-trauma center; PMFR: physical medicine and functional rehabilitation; HGEP: hepato-gastro-enterology and proctology; IDD: infectious disease department; DHCC: day hospital collecting center; ICU: intensive care unit

The patient's ages ranged between 1 day to 65 years, and the median age was 24.5 years old, with a gender distribution of 38/73 (52%) females and 35/73 (48%) males. The prevalence of infections was highest among patients of age-group 21-50 years 32/73 (44%) followed by < 2 months 17/73 (23%), < 20 years 11/73 (15%), and least 51-65 years 7/73 (10%). Data were not available for 6 patients. Bacteremia infections and respiratory tract infection (RTI) were highest in newborns and patients under 20 years old. In contrast, urinary tract infection (UTI) was predominant in patients aged 21-50 (Table 3).

**Table 3:** distribution of infections among gender and patients' age groups

Age	< 2 months	< 20 years	21-50	51-65	Unknown
**Gender**					
Female	9	8	12	6	3
Male	8	3	20	1	3
**Infections**					
Bacteremia	5	4	11	1	3
UTI	-	2	8	1	3
RTI	9	3	2	2	-
Ascites	-	-	1	-	-
Otitis	-	1	-	-	-
Surgical wound infection	3	-	5	-	-
Rectal carriage	-	1	5	3	-

HAI: health-associated infections; UTI: urinary tract infection; RTI: respiratory tract infections

**Bacteriological data:** for enterobacteria isolates, *E. coli* was the predominant (50%) 19/38, and *Klebsiella pneumonia* was the second predominant (31.6%) 12/38, and then *Enterobacter cloacae* (10.5%) 4/38, *Klebsiella oxytoca* (2.6%) 1/38, *Raoutella terrigena* (2.6%) 1/38, *Enterobacter aerogenes* (2.6%) 1/38.

**Antimicrobial susceptibility profile:** the resistance rates of 73 isolates were very high to most antimicrobials tested (Table 4). Of the 38 MDR isolates, 37/38 (97%) were ESBL-producers, 21/38 (55%) were ESBL/carbapenemase producers, and 1/38 (3%) carbapenemase producers. All enterobacteria (100%) were resistant to 4 antibiotics from three antibiotic classes. Most isolates (more than 90%) were resistant to amoxicillin clavulanic acid, cefepime, and ceftriaxone. In addition, a substantial level of resistance to quinolones and aminoglycosides was observed. In contrast, the lowest resistance rate was observed in gentamicin (29%). Regarding *A. baumannii*, the resistance rates were remarkably higher for all antibiotics tested; 84% of strains were XDR. Imipenem, meropenem, ciprofloxacin, levofloxacin, gentamicin, and tobramycin (100%); amikacin, netilmicin, and trimethoprim/sulfamethoxazole (more than 80%). All of *P. aeruginosa* were MDR and showed a high level of resistance to piperacillin/tazobactam and gentamicin (60% and 50% respectively), cefepime (70%), levofloxacin (80%), netilmicin, and tobramycin (100%). However, amikacin was the most active; the resistance level was 30%. The prevalence of resistance against the tested antibiotics is presented in Table 4.

**Table 4:** percentage antibiotics susceptibility of isolates

Antibiotics	Resistant *Enterobacteriaceae* % (n= 38)	Resistant *A. baumannii* % (n=25)	Resistant *P. aeruginosa* % (n=10)
Ampicillin	100	-	-
Amoxicillin/ clavulanic acid	92	-	-
Piperacillin/ tazobactam	63	-	60
Cefepime	97	-	70
Ceftazidime	100	-	40
Cefotaxime	84	-	-
Ceftriaxone	92	-	-
Meropenem	42	100	-
Imipenem	34	100	100
Ertapenem	61	-	-
Ciprofloxacin	76	100	60
Levofloxacin	45	100	80
Amikacin	34	96	30
Gentamicin	29	100	50
Tobramycin	100	100	100
Netilmicin	100	88	100
Fosfomycin	5	-	-
Trimethoprim/ sulfamethoxazole	82	84	-
Chloramphenicol	100	-	-

**Molecular resistance analysis of *Enterobacteriaceae* isolates:** after amplifying the target genes, PCR showed that all isolates harbored genetic support of the resistance. Of the 37 ESBL-producer, *bla*_CTX-M_ were the mainly detected gene (65% 25/37), eight strains harbored *bla*_SHV_ (22%; 8/37) and (11%; 4/37) carried *bla*_TEM_. Of the 22 carbapenemase-producers, 9 were positive to *bla*_OXA-48_, 4 for *bla*_NDM_, VIM gene was not detected in any of our isolates. Furthermore, the coexistence of *bla*_CTX-M_with *bla*_OXA-48_ and *bla*_CTX-M_ with *bla*_NDM_ was detected in 2 strains, the coexistence of *bla*_CTX-M_ with *bla*_SHV_ and *bla*_OXA-48_ was detected in one strain, 4 strains harbored *bla*_SHV_ with *bla*_OXA-48_. Of the 9 cases positive to *bla_OXA-48_*, six have been reported in an isolate of *K. pneumoniae*, two in *E. coli* and one of *E. cloacae*. This distribution of the mentioned genes among the enterobacteria species is shown in Table 5.

**Table 5:** distribution of genes mediated resistance among *Enterobacteriaceae* species

Genes	CTX-M	SHV	TEM	OXA-48	NDM	CTX-M /OXA-48	CTX-M/NDM	CTX-/SHV/OXA-48	SHV/XA-48	SHV/NDM
**Species**
*E. coli*	12	-	2	-	1	2	1	-	-	1
*K. pneumoniae*	5	1	-	1	-	-	-	1	4	-
*E. cloacae*	-	1	2	-	-	1	-	-	-	-
*K. oxytoca*	1	-	-	-	-	-	-	-	-	-
*E. aerogenes*	-	-	-	-	-	-	1	-	-	-
*R. terrigena*	1	-	-	-	-	-	-	-	-	-

## Discussion

Due to the overuse of antibiotics, invasive procedures, and devices expose patients to a high risk of comorbidity and mortality. Therefore, in developing countries such as Morocco, investigating and controlling bacterial infections' epidemiology and resistance patterns is critical, especially in hospitals, for clinicians and decision-makers on health to implement adequate measures and effective management of these diseases [15].

In the current primary descriptive study, we examined the epidemiological spectrum by evaluating the antimicrobial patterns of the gram-negative bacilli clinical isolates of patients hospitalized in various wards of University Hospital Center Ibn Rochd of Casablanca, Morocco. Our study revealed that the most common culture-positive clinical samples were blood followed by urines. Similar findings were obtained in several studies [16,17]. However, in the investigations carried out by Beyene *et al*. and Mirzaei *et al*. urinary infections were the predominant, followed by bacteremia [18,19]. It is important to note that our study's demographics data showed that bacteremia and respiratory tract infections affect mainly infant patients <2 months old due to their lack of fully developed immune status. This result differs from published Moroccan and international epidemiology, ranging from 20 to 67 years old [20,21]. The detected difference could be due to the brief period of study, the diversity of specimen type, and specimen size. In the specified period, 38 *Enterobacteriaceae* MDR were isolated, *E. coli* and *K. pneumonia* were the most frequent species, mainly in bacteremia and urinary tract infections. Studies have shown the prevalence of these micro-organisms in these infections in Iran, Brazil, and Nigeria [17,22,23].

On the other hand, non-fermenting gram-negative bacilli are represented essentially with *A. baumannii* and *P. aeruginosa* plays a key role in nosocomial infections. At the same time, they were only considered to be contaminants in the past. Our study isolation rate of *A. baumannii* and *P. aeruginosa* imipenem resistant was 34% and 14%, respectively, parallel to those reported in the literature [19,20]. In this study, *A. baumannii* was isolated mainly from blood cultures and medical equipment, from ICU, especially neonatology which is the most alarming. This finding is in agreement with many reports [17,20,21,24,25]. According to a study conducted in the United Kingdom, *Acinetobacter spp* stains causing bacteremia have increased between 1998 and 2005 from 2 to 18 per 100,000 hospital days [26]. Our study observed a higher incidence of isolates from ICU (47%). Several risk factors account for the high ICU infections. These first ICU houses critically ill patients who are often exposed to extensive use of antibiotics, causing selection pressure for the emergence of resistance. These factors coupled with interventional instrumentations such as mechanical ventilation and invasive procedures commonly used expose the patients to a high risk of infections.

Antimicrobial resistance has become a global pandemic that threatens every individual's health, economic and social well-being. The current prioritized lists of bacterial pathogens by the World Health Organization and the Infectious Diseases Society of America categorized *E. coli, E. cloacae, K. pneumoniae, A. baumannii*, and *P. aeruginosa* have further been described as gram-negative *Enterococcus faecium, Staphylococcus aureus, Klebsiella pneumoniae, Acinetobacter baumannii, Pseudomonas aeruginosa*, and *Enterobacter* species (ESKAPE) as critical or most life-threatening gram-negative pathogens [27].

The susceptibility profile of the isolates displayed a high level of multi-drug resistance against the first-line drug such as penicillin, cephalosporin, fluoroquinolones due to the unrestricted use by physicians, in addition, to their purchasing ease without prescription in the community pharmacies. Furthermore, isolates showed a high rate of resistance to carbapenem. The antibiotic resistance pattern of *Enterobacteriaceae* showed 100% of resistance to ampicillin, tobramycin, netilmicin, and chloramphenicol; more than 80% of the strains were resistant to amoxicillin/clavulanic acid, cefepime, cefotaxime, ceftriaxone, and trimethoprim/sulfamethoxazole, which is similar to previous studies [18,22]. Likewise, high-rate resistance against piperacillin/tazobactam (63%), ertapenem (61%), and ciprofloxacin (76%) was observed; this may be due to the coexistence of other mechanisms of resistances as inactivating enzymes like β-lactamases, carbapenemases, and aminoglycosidases. The widespread of these bacterial profiles in Morocco is a common trend in hospitals [20,28], community [29], and even the environment [30]. However, fosfomycin (for *E. coli*), amikacin, and gentamicin were the most active antibiotics. The resistance to aminoglycosides is usually attributable to enzymes modification, such as amino-glycoside *6´-N*-acetyltransferases of type Ib (AAC (6')-Ib). This enzyme can modify amikacin, tobramycin, kanamycin, and netilmicin but not gentamicin. Numerous studies reported the existence of the *aac(6’)*-Ib gene and susceptibility to amikacin and gentamicin [31].

Resistance to antibiotics tested in *A. baumannii* in the current study reached very alarming levels and were screened as follow: imipenem (100%), meropenem (100%), ciprofloxacin (100%), levofloxacin (100%), gentamicin (100%), and tobramycin (100%). In addition, up to 96%, 88%, and 84% of isolates were resistant to amikacin, netilmicin, and trimethoprim/sulfamethoxazole. The infective of carbapenems, even if they're used as a last resort, poses a challenge. The results present similarity with increasing rates of resistance of *A. baumannii* around the world, in Iran [19], Nepal [32], and Morocco [20,25], Although this rate seems to increase since 2007 in Moroccan hospitals. Indeed, Lahsoune *et al*. reported a resistance rate of 17.9% and 35% to netilmicin and amikacin [33]. In 2016, Uwingabiye *et al*. reported that resistance to the same antibiotic increased to reach 33% and 52% [34]. Overall, the variation in antibiotic resistance proportion may be due to many parameters such as geographical difference, study period, and ward origin.

In addition, the overall resistance of *P. aeruginosa* against aminoglycoside, cephalosporin, and fluoroquinolones was alarming, for netilmicin (100%), tobramycin (100%), cefepime (70%), levofloxacin (80%), and ciprofloxacin (60%). However, it was observed that amikacin and meropenem's resistance rate by these isolates was not that high. Amikacin and Meropenem have been reported to effectively treat diseases caused by *P. aeruginosa* [35]. In a study conducted at the Children's Hospital of Tehran between 2013 and 2018, showed that the sensitivity of *P. aeruginosa* has increased from 50% to 76% [17]. Therefore, the susceptibility of amikacin may be due to its use as a last resort in treating severe infections.

In general, the international literature reports that the resistance to extended-spectrum β-lactams in *Enterobacteriaceae* is mainly mediated via the production of *bla*_CTX-M_ and to a lesser degree via *bla*_SHV_ [36]. In most local studies, carbapenem resistance in *Enterobacteriaceae* is often mediated by the production of OXA-48 and NDM carbapenemases. Therefore, this outspread of the OXA-48 enzyme could be endemic for several Mediterranean countries, such as Morocco's case [37-39]. This increasing emergence is a severe epidemiological risk, as it is well known that plasmid-mediated transfer of drug-resistance genes is considered to be one of the most essential mechanisms driving their spread. Also, the strains that carried such genes cause nosocomial infections and outbreaks with a high mortality range. Thus, available choices for appropriate treatments for infections are very limited and that´s why the lack of their detection may enhance their hidden. Thus, it is essential to conduct molecular genetic analyses as well as to characterize the β-lactamase and carbapenemase found in specific geographic areas to outcome of the molecular evolution of both genes and resistant clones.

**Limitations:** our investigation´s main limitations were, first of all, the short duration of the study period, which led to a small sample size also related to inclusion criteria. Secondly, it is a single-centered study. However, the scope of this study was to investigate the resistance pattern and prevalence of MDR and XDR among the studied groups of pathogens in our hospital. We would also have wished to detect the genes coding carbapenemase for *A. baumannii* and *P. aeruginosa*.

## Conclusion

Gram-negative bacteria are one of the leading causes of significant antibiotic resistance. The *Enterobacteriaceae, A. baumannii* and *P. aeruginosa* isolated during this study were implicated in various infections. The bacteremia was the most frequently diagnosed, also notably, the infections were common among infants. Our results confirm, on the one hand, the international trend of increasing multi-drug resistance among gram-negative bacilli. Withing the *Enterobacteriaceae*, 55% were ESBL, 42% were ESBL and carbapenemase producing, while 3% were carbapenemase, in addition to the coexistence profile to the several antibiotic classes. Regarding nonfermenting gram-negative bacilli, 84% of *A. baumannii* were extensively drug-resistant, while all *P. aeruginosa* were MDR. On the other hand, the study underlines the local spread of *bla*_CTX-M_, *bla*_OXA-48_, and *bla*_NDM_. Nevertheless, the spread of these genes in our isolates, with the coexistence of genes mediated resistance, could be further than expected and lead to a management impasse. Given that antibiotic resistance can cause severe infections in hospitalized patients and lead to a therapeutic dilemma, these findings highlight the need for timely, accurate epidemiological and susceptibility information to guide the identification and implementation of strict antimicrobial stewardship policies and good microbiological surveillance procedures in the hospitals.

### What is known about this topic

- *The increasing antibiotic-resistant infections area worldwide dilemma, which is a significant cause of mortality and morbidity;*
- *The spreading of drug resistance among bacteria is affected by many parameters and may differ in different parts of the world;*
- *In lower-middle-income countries, resistance rates are high*.

### What this study adds

- *This study is showing a recent resistance and epidemiological profile among gram-negative isolates, as well as their spreading pattern in the various wards of University Hospital Center Ibn Rochd of Casablanca, Morocco;*
- *A higher bacteremia and respiratory tract infections rate, that affects infant patients;*
- *The genotypic approach used to detect the molecular resistance mechanism among the Enterobacteriaceae isolates, shows mainly the spreading of bla_CTX-M_ and bla_OXA-48_, in addition to various profiles of coexisted genes*.
